# Microsurgical resection of foramen magnum meningioma through a transcondylar approach: three-dimensional operative video

**DOI:** 10.3171/2019.10.FocusVid.19465

**Published:** 2019-10-01

**Authors:** Guilherme H. W. Ceccato, Rodolfo F. M. da Rocha, Duarte N. C. Cândido, Wladimir O. Melo, Marcio S. Rassi, Luis A. B. Borba

**Affiliations:** 1 School of Medicine, Federal University of Paraná, Curitiba, Paraná, Brazil;; 2 School of Medicine, Faculdade Evangélica do Paraná, Curitiba, Paraná, Brazil;; 3 Department of Neurosurgery, Santa Casa de Misericórdia de Maceió, Maceió, Alagoas, Brazil;; 4 Department of Neurosurgery, Hospital Monsenhor Walfredo Gurgel, Natal, Rio Grande do Norte, Brazil;; 5 Department of Neurosurgery, Evangelic University Hospital of Curitiba, Paraná, Brazil; and; 6 Department of Neurosurgery, Federal University of Paraná, Curitiba, Paraná, Brazil

**Keywords:** foramen magnum, meningioma, transcondylar, brainstem, craniovertebral, video

## Abstract

Foramen magnum (FM) meningiomas are challenging lesions. We present the case of a 38-year-old female with neck pain, dysphonia, and slight twelfth nerve palsy. Imaging workup was highly suggestive of an FM meningioma, and microsurgical resection with the aid of intraoperative neurophysiological monitoring was indicated. A transcondylar approach was employed, the vertebral artery was mobilized, and the tumor was completely removed. Postoperative MRI demonstrated complete resection. There were no signs of cervical instability. The patient presented with improvement of her symptoms and no new neurological deficit on follow-up. FM meningiomas can be successfully resected using a transcondylar approach, since it increases the exposure of the ventral FM, allowing the surgeon to work parallel to the skull base and flush with the tumor’s attachment. Informed consent was obtained from the patient for publication of this operative video.

The video can be found here: https://youtu.be/itfUOB-6zM0.

## Transcript

### 0:20 Presentation and clinical history

This is a three-dimensional operative video of the microsurgical resection of foramen magnum meningioma through a transcondylar approach. The patient was a 38-year-old female presenting a history of neck pain, dysphonia, and a slight XII nerve palsy.

### 0:35 Preoperative imaging

Preoperative MRI demonstrated a well-delimited lesion highly suggestive of foramen magnum meningioma, with significant compression of the neural structures. Comparing pre- and postcontrast T1-weighted image we observe a homogeneous enhancement of the lesion after contrast administration.

In the FIESTA sequence we note a CSF layer between tumor and the brainstem that corresponds to an arachnoid dissecting plane (Bruneau and George, 2008).

### 1:00 3D models

In this 3D model we can better understand the lesion and see its important mass effect over the cervicomedullary junction and the close relation with the vertebral artery. Here we can see the region that will be approached and the bone structures that will be removed. A suboccipital craniotomy limited laterally by the sigmoid sinus will be performed associated with the C1 hemilaminectomy (Borba et al., 2009). And we should note that in this step also the vertebral artery is released from the foramen transversarium, in order to allow mobilization of the vessel away from the condyle (Wen et al., 1997; Bruneau and George, 2008). After that a part of the posterior portion of the occipital and atlantal condyles is removed, increasing the exposure of the ventral foramen magnum (Wanebo and Chicoine, 2001; Muthukumar et al., 2005; Rhoton, 2000).

### 1:39 Anatomy

In these anatomical pictures we review the steps of the procedure, but the approach that will be employed in this case is more lateral than in this anatomical demonstration. Here we see the trapezius muscle, the sternocleidomastoid, and splenius capitis. Underneath the trapezius and sternocleidomastoid, we see the splenius and we start to see the semispinalis capitis. Reflecting the splenius, we look better the semispinalis capitis and observe the longissimus muscle. Also, the occipital artery is seen. The suboccipital triangle is finally reached after reflecting the semispinalis capitis. The boundaries of this area are formed by the superior oblique, the inferior oblique, and the rectus capitis posterior major. Inside it we find the vertebral artery, surrounded by a dense venous plexus called the suboccipital cavernous sinus. In this lateral perspective we observe the longissimus capitis after reflecting the splenius, and note the muscles related to the suboccipital triangle deeper. Reflecting the muscles that form the suboccipital triangle we observe the vertebral artery emerging from the transverse process of C1 and coursing behind the atlantal condyle. And here we have a close view of the vertebral artery and the atlantal and occipital condyles. In this image we have the exposure following a suboccipital craniotomy and C1 hemilaminectomy, and now the dural opening. Here we see the hypoglossal nerve entering in the hypoglossal canal and the third and fourth segments of the vertebral artery. In this view we observe the hypoglossal nerve and canal, and the jugular foramen related to the glossopharyngeal, vagus, and spinal accessory cranial nerves. Here we have an anatomical dissection comparing the exposure following a far lateral and transcondylar approach. We note the greater exposure provided after resecting the condyle, that can block the view of the ventral foramen magnum. By drilling the condyle we can look in front of the cervicomedullary junction and even see the contralateral vertebral artery. Here we have a demonstration of the IX, X, and XI nerves in the jugular foramen, and the hypoglossal nerve coursing inside the canal (Rhoton, 2000; Wen et al., 1997).

### 3:44 Patient positioning and incision

The patient was placed in a lateral position, with the head parallel to the floor, with the ipsilateral shoulder slightly displaced anterior and inferiorly. Skin incision was carried in C-shape, centered in the mastoid tip, initiating around 3 cm behind the level of pinna and running posteriorly until C4 (Rassi et al., 2017).

The skin is incised and reflected. We see the sternocleidomastoid muscle and the great auricular nerve. The sternocleidomastoid is detached and reflected, and we can see underneath the splenius capitis that is also detached and displaced. Here we demonstrate the longissimus capitis that is mobilized. And the suboccipital triangle is better seen here, with the vertebral artery inside, and we note the superior and inferior obliques.

### 4:27 Procedure

These muscles are displaced, with bleeding coming from the suboccipital cavernous sinus easily controlled. Soft tissues are dissected, exposing the occipital condyle, C1 arch, and the vertebral artery. The condyle starts to be drilled, and also the ipsilateral half of the C1 arch, releasing the artery from the foramen transversarium. Bleeding coming from the venous plexus is controlled with fibrin glue. The suboccipital craniotomy is performed with the drill and the bone flap is removed. The posterior portion of the occipital condyle continues to be drilled, with bleeding coming from the posterior condylar emissary vein easily controlled (Borba et al., 2009). Drilling continues until to be finished and we observe the potential gain of exposure when retracting dura over the space created (Borba et al., 2009; Barut et al., 2009; Wen et al., 1997). Some last adhesions of the vertebral artery are sectioned to be freed extradurally. Here the dura starts to be opened. And we can already see some part of the tumor. We see the entry point of the vertebral artery in the dura and the dural opening continues carefully in order to free the artery from its dural attachment. Here we see the dentate ligament. Now we observe the close relation of the tumor with the vertebral artery, but existing a good cleavage plane between them. Thanks to that, adhesions between the tumor and the artery are cut and the vessel is progressively released. Then gradually the artery is being detached from the surrounding tumor. Now the third and fourth segments of the vertebral artery can be mobilized, with the vessel freed intra- and extradurally. The lesion surface is initially coagulated to minimize bleeding and then the resection starts at the base of tumor, in order to initially attack its blood supply. After disconnecting the tumor from its attachment in the foramen magnum, careful traction exposes the lesion and here we observe the contralateral vertebral artery and here the ipsilateral vessel. Then we perform the tumor debulking, decompressing the adjacent cervicomedullary junction. Then the lesion is removed. Here a remaining part of the tumor closely related to the cervicomedullary junction is resected, and we note the proximity of the vertebral artery. Here we observe the important gain of exposure provided by condylar drilling. Now we are looking from the bottom up and we start to remove the tumor around the hypoglossal nerve and decompress it. Then progressively the lesion involving the hypoglossal nerve is dissected and removed, relieving the compression over the nerve. The site of tumors attachment is dissected, coagulated, and careful section of adhesions is employed. Here we observe the hypoglossal nerves from both sides (Wen et al., 1997). Coagulation of the site of tumors attachment in the dura is performed, and the piece related to the hypoglossal nerve is removed. Now a small piece of tumor that extended toward the jugular foramen is resected. We can observe the vertebral artery completely freed, the spinal accessory nerve, and the hypoglossal nerve completely decompressed. Now we look to the jugular foramen. We inspected the surgical cavity, here the region of the jugular foramen. And we can see now the basilar artery arising after joining both vertebral arteries. Here we see the contralateral hypoglossal nerve.

### 9:19 Closure

In the closure the condyle defect region was covered with the superior oblique muscle. A watertight dural closure using fascia lata was performed and the muscles were brought back for the anatomical position (Rassi et al., 2017).

### 9:33 Postoperative imaging

Postoperative MRI demonstrates complete tumor resection, with decompression of the cervicomedullary junction. Comparing the CT scans prior and after surgery we can observe the importance of condyle drilling in this case. It enhanced the surgical exposure and minimized manipulation of the neural structures (Wanebo and Chicoine, 2001).

### 9:52 Outcome

Pathology confirmed the lesion to be a WHO grade I meningioma. The patient presented improvement of symptoms, with no new neurological deficits in the follow-up, and no dysphagia or dysphonia was observed. Here we demonstrate preservation of tongue motility, with preservation of the hypoglossal nerve, and also demonstrate preservation of facial expression. We emphasize that in this case there was no need for occipitocervical fusion, and no craniocervical instability was observed.
